# Effect of Sampietrini Pavers on Urban Heat Islands

**DOI:** 10.3390/ijerph182413108

**Published:** 2021-12-12

**Authors:** Laura Moretti, Giuseppe Cantisani, Marco Carpiceci, Antonio D’Andrea, Giulia Del Serrone, Paola Di Mascio, Giuseppe Loprencipe

**Affiliations:** 1 Department of Civil, Constructional and Environmental Engineering, Sapienza University of Rome, Via Eudossiana 18, 00184 Rome, Italy; giuseppe.cantisani@uniroma1.it (G.C.); antonio.dandrea@uniroma1.it (A.D.); giulia.delserrone@uniroma1.it (G.D.S.); paola.dimascio@uniroma1.it (P.D.M.); 2 Department of History, Representation and Restoration of Architecture, Sapienza University of Rome, Piazza Borghese 9, 00186 Rome, Italy; marco.carpiceci@uniroma1.it

**Keywords:** urban heat island, albedo, emissivity, cool pavements, sampietrini, permeability, air temperature

## Abstract

Cool pavements are reflective and/or permeable pavements that improve microclimate of urban areas where heat islands cause discomfort to citizens. Stone pavements lower surface temperatures and reduce the amount of heat absorbed. This study assessed, using ENVI-met 4.3 LITE software, how air temperature and predicted mean vote depend on physical properties of the road pavement. A comparative microclimatic analysis was implemented on a rectangular square in Rome (Italy) in the summer, paved in three different ways: asphalt, traditional sampietrini, and permeable sampietrini. The model considered local weather parameters, surrounding fabric, and vegetation to give reliable results in terms of numerical and graphical output using the application tool Leonardo. The tested pavement types affected air temperature during the day, but did not influence this variable in the early morning. Permeable sampietrini pavement was more effective than traditional sampietrini pavement in reducing air temperature compared to the current asphalt surface. The road pavement did not, however, affect human comfort in terms of predicted mean vote. The obtained results are useful for further investigation of parameters that could modify the microclimatic conditions of urban areas.

## 1. Introduction

In the last few years, the effects of current global warming (e.g., rise of mean surface temperature, heating of the oceans and mass loss from glaciers and ice caps), climatic variability, and extremes of weather phenomena, have been increasing [1,2,3]. Human activities are one of the main causes that contribute to extreme climate events [4,5,6] and extreme heat events (e.g., heat waves) are one of the most serious and harmful [7,8]. A heat island is caused by anthropogenic forcing from land cover use: it substitutes natural surfaces with impermeable ones and builds large obstacles that absorb heat during the day and release it during the night [9]. Such conditions lead to urban heat islands (UHI) where temperatures are 2 to 15 °C warmer than those in rural and surrounding areas [10,11,12] due to the change in albedo. This negatively affects both environment and livability because of increase of the energy demand and emissions to the air [13,14]. The main elements affecting differences between urban and rural spaces are the presence of green areas and the materials used in urban areas (e.g., asphalt for roads and concrete for buildings) [9,15]. The physical properties of urban building materials lead to heating of the urban fabric. In particular, asphalt road pavements play a pivotal role in the formation of UHIs [9,16] with up to 40% of urban surfaces potentially covered with asphalt [17]. Asphalt has low albedo value (i.e., the ability of a material to reflect solar energy), which affects maximum temperatures, and a high emissivity value (i.e., the ability of a material to radiate energy or, rather, to emit energy by radiation [13]), which instead influences minimum temperatures [13,18]. The darker a material, the lower is its albedo [14,19]; lighter colored materials are to be preferred. Whatever the color, impermeability of pavement materials should be avoided to allow evapotranspiration from water infiltration in the subsoil [9]. In addition, the paucity of green areas in urban areas reduces the benefits of evapotranspiration, thus increasing air temperature [15]. In order to mitigate UHI, urban green infrastructures (UGI) (e.g., green roofs, green walls, green corridors, and green networks) can be designed [20] and added to traditional green areas (e.g., parks and urban woodland). UGI are less invasive than the construction of new green areas in the city center where buildings and road network affect the opportunity to design new green areas. These alternatives consist of covering roofs and walls with a vegetal layer that increases the evapotranspiration process and contributes to cooling of the environment by both reducing air temperatures and reducing surface temperatures of the buildings, thus improving quality of life [5,20].

To counter the tendency to UHI, new surfaces have been introduced. For example, cool pavements can mitigate the key problems associated with traditional asphalt [21,22,23]. Cool pavements can be divided into two main categories [5,9,11,24,25]:

1. Reflective pavements. Their high albedo value contributes to reducing the surface temperature. An increase in the albedo value can be obtained by modifying two critical parameters: the color and the roughness of the pavement. Making the surface lighter or smoother decreases the amount of heat that it absorbs and instead increases the amount of reflected heat. Thus, high reflectance light paints and lighter building materials (e.g., clear concrete and stone materials) could lower surface temperatures in urban areas [26]. This complies with the approach taken by the Italian Ministry of the Environment [27] that requires “cool” pavements (e.g., white stones, permeable concrete blocks, paving grids for grass parking lots) to prevent UHI.
2. Porous pavements. These have higher porosity than conventional pavements. The voids allow the entry of floods into the pavements and water infiltrated below the pavement evaporates while the surface heats up. The obtained evaporative cooling of the pavements counters the heat island effect. The performance of a permeable pavement can be assessed considering three criteria: the ability to reduce the surface temperature by evaporation, the ability to reduce the risk of flooding, and pavement durability [26].

Stone pavements are among the most effective for cooling. They are made of stone materials, mainly of light color, and their laying pattern ensures permeability due to voids between blocks [28,29]. Modular stone pavements, for example, are both reflective and permeable. Stone pavements are made of blocks of various sizes and types:

1. pebble pavements are composed of river stone elements,
2. flagstone pavements are composed of parallelepiped blocks of stone, in which the plant dimensions are higher than the width of the elements,
3. cobblestone pavements are composed of parallelepipeds, in which the three dimensions have comparable magnitudes.

**Figure 1:**
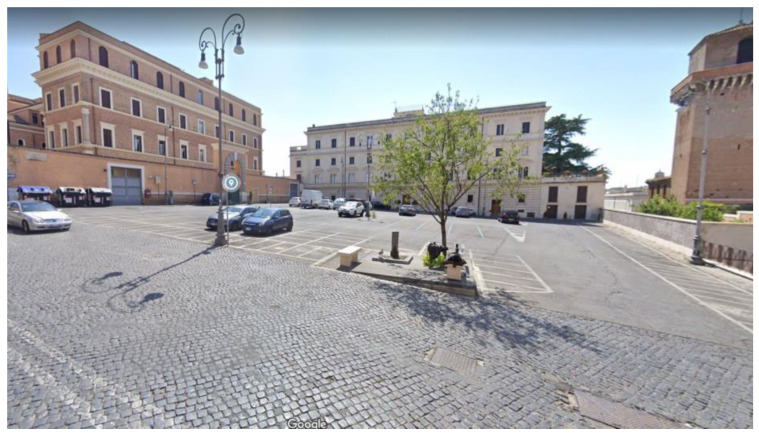
St Peter in Chains’ square, Rome, Italy (photo: Google maps).

This study investigated how the microclimatic conditions of an urban area vary by modifying the existing asphalt pavement in order to reduce UHI. The dynamic simulation tool ENVI-met 4.3 LITE [30,31] allowed comparative analysis of various mitigation strategies towards the UHI effect. Sampietrini pavements with traditional and permeable patterns were modeled to assess the air temperature and the human sensation of thermal comfort in a square in the Monti neighborhood in Rome (St Peter in Chains’ square, Figure 1) during the hottest summer days. The area is in the historic center of Rome, where modifications to counter the heat island effect can affect only the road pavement due to the urban constraints.

The obtained results allowed an objective comparison between the microclimate performances of common urban pavements to achieve the objectives of the international effort to prevent damage from climate change.

## 2. Materials and Methods

Albedo and emissivity are the main factors affecting the surface temperature of road pavements [13]. Albedo ranges between 0 and 1, where 0 is an ideal black surface without reflection that absorbs all incident radiation and 1 corresponds to a white surface that reflects all incident radiation (maximum reflection) [22]. The emissivity of a material is the fraction of energy radiated by that material compared to the energy radiated by a black body at the same temperature. It is a measure of a material’s ability to radiate energy. Emissivity ranges between 0 and 1 and quantifies the effectiveness in emitting energy as thermal radiation; a true black body has emissivity equal to 1, while any real object has an emissivity between 0 and 1 (gray body). Therefore, these variables represent criteria for choosing a pavement to counter UHI [13]. Building materials currently used to pave roads have high emissivity and low albedo coefficients [13,19,32] (e.g., conventional asphalt pavements can reach up to 48–67 °C surface temperatures [13]). The best pavement materials should, instead, have a high value of albedo and a low value of emissivity. In road construction, stone materials such as porphyry, basalt, granite, and compact limestone are the most frequently used to build cool pavements. Table 1 lists the emissivity and albedo coefficients of traditional and cool building materials [33,34]. The values in Table 1 show that asphalt, one of the most common pavement materials, is the worst with respect to urban heating.

**Figure 2:**
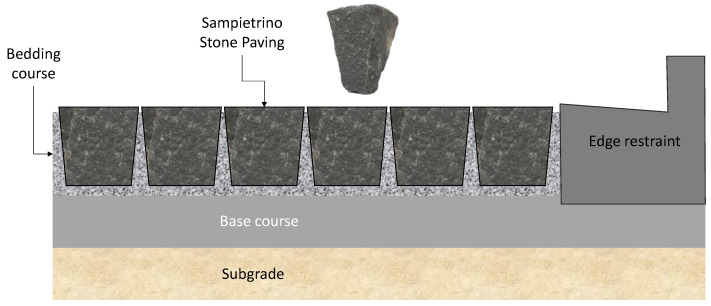
Cross section of a sampietrini pavement.

According to Table 1, the emissivity coefficients of asphalt and stone materials are comparable, but their albedo significantly differs and warrants this study on sampietrini cobblestones, a historic pavement common in the urban road network of Rome. Black basalt pavers named sampietrini [35,36] have a cubic or truncated-cone shape whose dimensions are approximately 12 × 12 × 18 cm and constitute the surface layer of a pavement (Figure 2).

**Figure 3:**
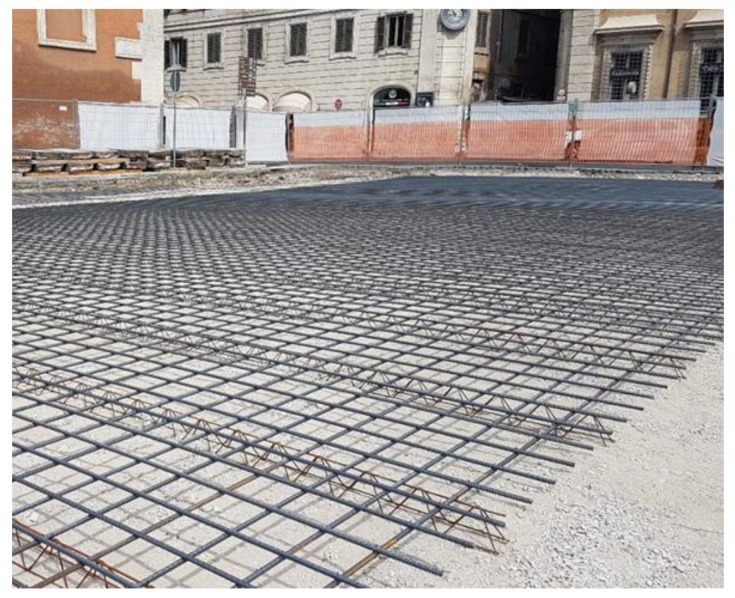
First phase: construction of reinforced concrete base course.

**Figure 4:**
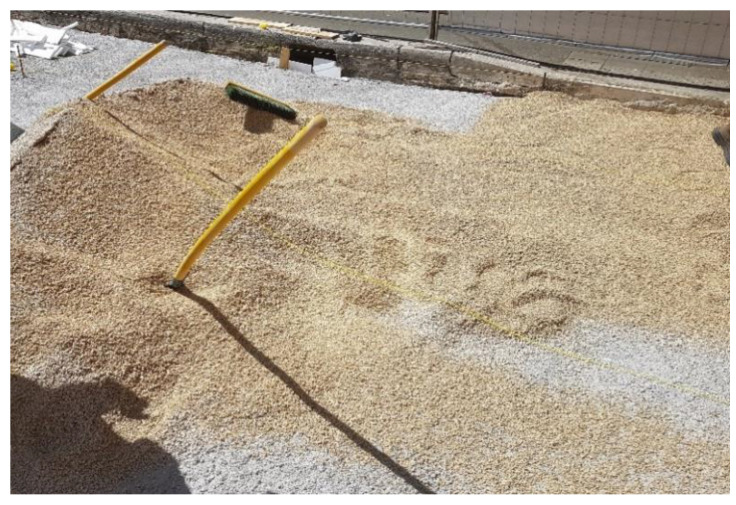
Second phase: construction of the bedding course.

**Figure 5:**
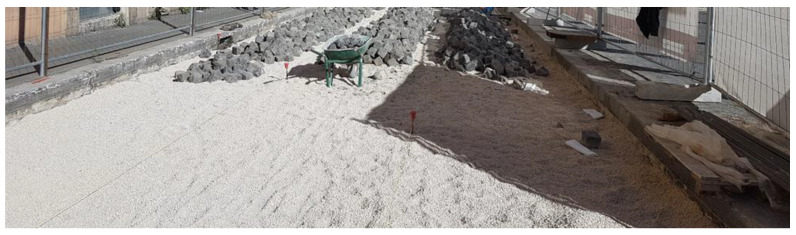
Second phase: dry laying of sampietrini.

**Figure 6:**
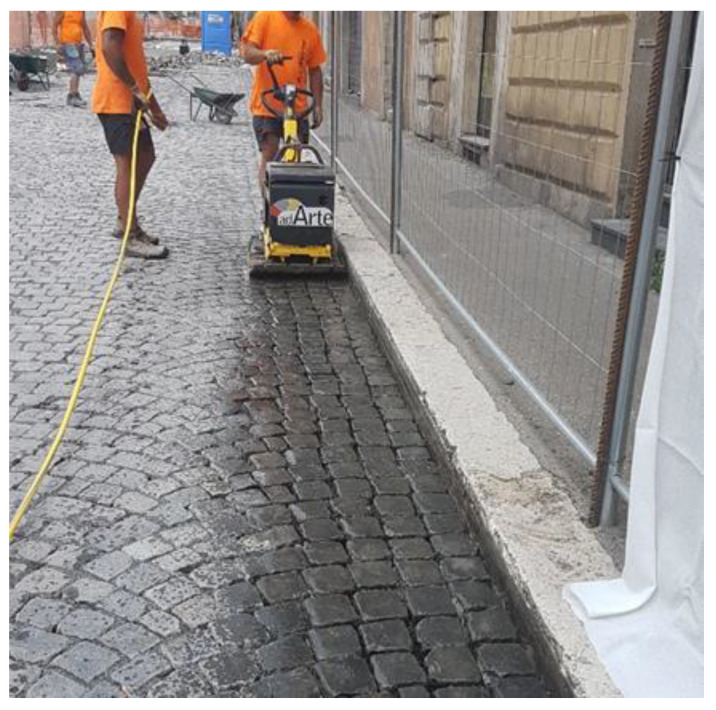
Third phase: sampietrini compaction.

**Figure 7:**
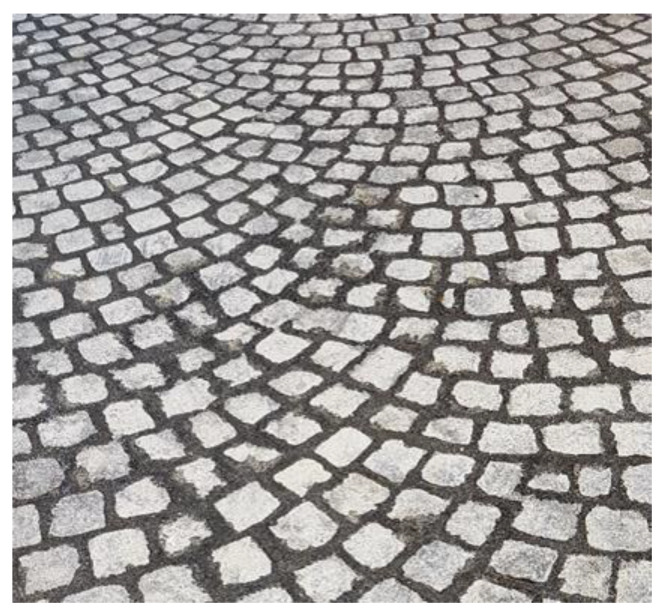
Fourth phase: sampietrini installation.

Their laying requires four steps:

1. first step: construction of reinforced concrete base course. On its impermeable surface water runs off and is then collected in retention basins (Figure 3).
2. second step: construction of the bedding course composed of sand according to the standard UNI 11714-1 [37]; pavers are then dry hammered into the sandbed (Figure 4 and Figure 5).
3. third step: compaction of the basalt surface using hand compactors (Figure 6).
4. fourth step: joint sealing with asphalt mastic (Figure 7).

**Figure 8:**
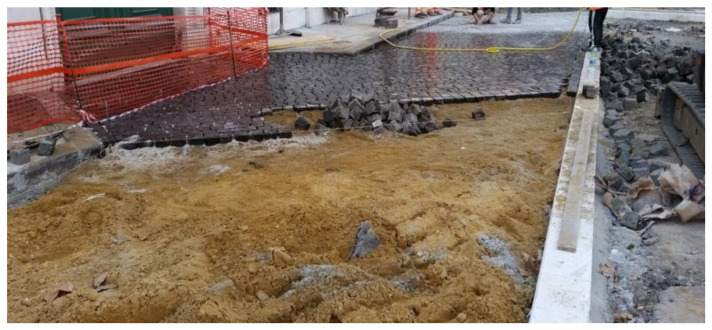
Sampietrini pavement under construction.

Figure 8 summarizes all laying phases of a sampietrini pavement.

**Figure 9:**
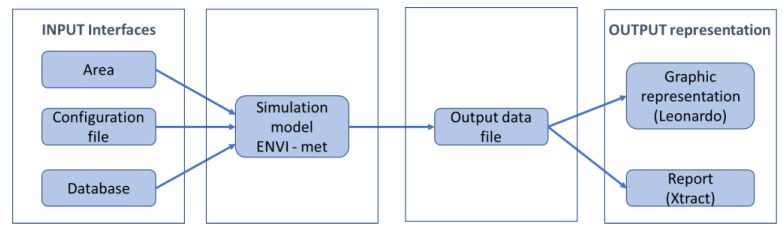
ENVI-Met layers.

The software ENVI-Met 4.2 LITE (ENVI-met GmbH, Essen, Germany) [28] enables microclimatic analysis providing a detailed calculation of parameters affecting the urban thermal environment [38]. The software is organized in four layers (as shown in Figure 9). There are three main input interfaces which converge in the simulation model that carries out the global analysis, applying the computational models, and returns the output files. The results can be read and analyzed through two reading outputs: Leonardo provides a graphical interface for displaying and analyzing numerical data of the physical results (e.g., air temperature and surface temperature) while Xtract allows extraction of binary output files to ASCII files.

Once the area under study is defined, it is divided in cells to form a grid; and greater the number of cells, the better is the degree of accuracy achieved. The model considers the local weather parameters, different types of pavement surface material, building layout, vegetation, and soil type to produce realistic results. All this information must be entered manually by defining temperature and wind speed and direction, as with other approaches [39]. The output files are divided into different themes, such as atmospheric, soil, and surface data, and are read through the Envi-met Leonardo interface. Each cell has a color that defines its specific condition (e.g., air temperature, specific humidity, mean radiant temperature, temperature cloths, and predicted mean vote) and colored maps are produced, as shown later.

Different scenarios were simulated considering an asphalt pavement and two sampietrini pavements; the former is standard, the latter is permeable. The modeled modular pavements differ with respect to in-between spaces and joint filler; in the permeable solution the laying pattern has wider joints than the traditional one [40,41] and the joints are filled with a water-permeable polymer sealing.

Table 2 lists the albedo and the emissivity coefficients of asphalt and sampietrini.

**Figure 10:**
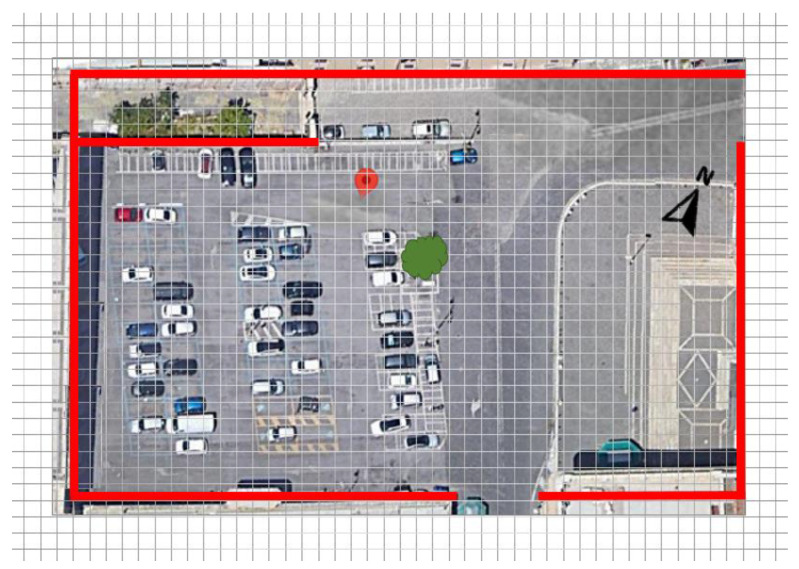
ENVI-Met model of St Peter in Chains’ square with grid and boundaries.

The study focused on St Peter in Chains’ square (lat. 41°53′36.8″–lon. 12°29′32.1″) where there are two road pavements (Figure 10). In the West sector, there is an asphalt pavement and in the East sector, there are sampietrini traditional pavers. The current layout was modeled with ENVI-Met with grid resolution 2 m × 2 m × 2 m (x, y, z). The ENVI-met model was composed of a one-dimensional (1D) boundary model, a main three-dimensional (3D) model, and a soil model.

**Table 2:** Physical characteristics of pavement materials.

Material	Albedo	Emissivity	Color in Figure 11, Figure 12 and Figure 13
Asphalt	0.20	0.90	
Sampietrini (traditional pattern)	0.40	0.90	
Sampietrini (permeable pattern)	0.40	0.92	

The building layout of the survey site was modeled, and three scenarios were analyzed.

**Figure 11:**
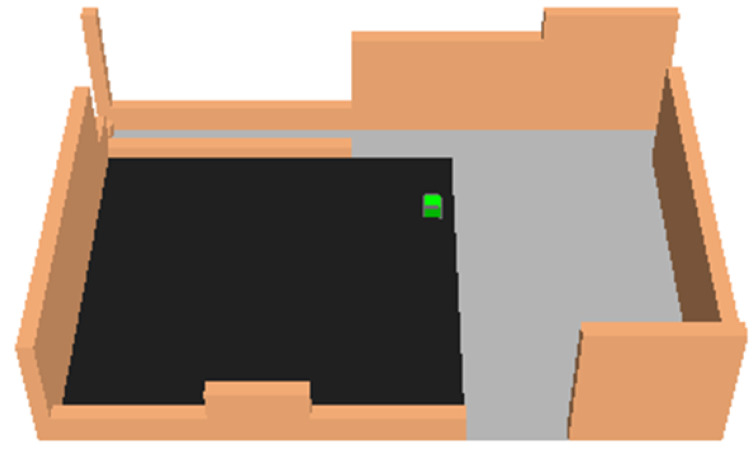
Current layout (S1).

**Figure 12:**
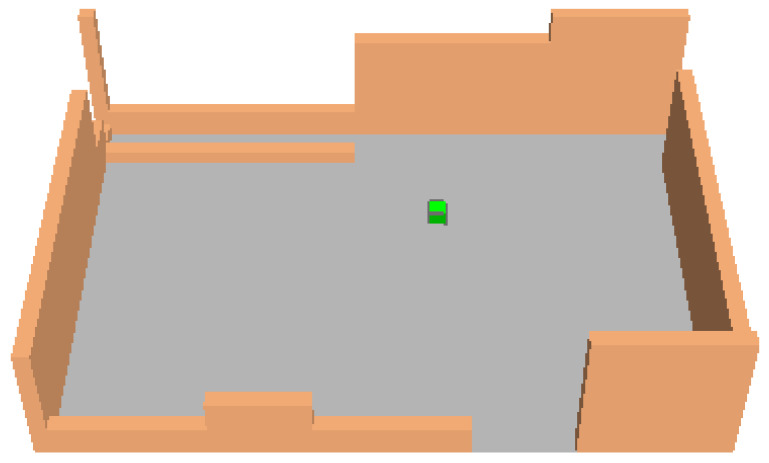
Traditional sampietrini pavement over all the square (S2).

**Figure 13:**
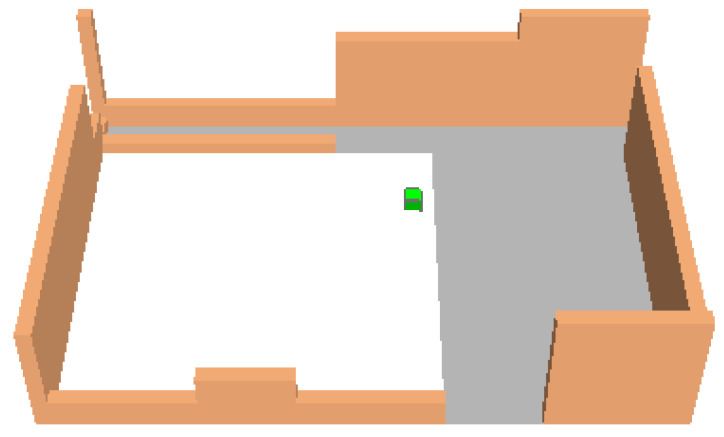
Permeable and traditional sampietrini pavement (S3).

1. first scenario (S1): the current layout, where asphalt and sampietrini compose the square pavement (black and dark gray areas in Figure 11, respectively).
2. second scenario (S2): traditional sampietrini pavement substitutes asphalt surface and covers all the square (Figure 12).
3. third scenario (S3): permeable sampietrini pavement substitutes asphalt surface in the square (white area in Figure 13).

Table 3 lists the input meteorological data, including maximum and minimum air temperature (Tmax and Tmin, respectively), wind speed, and wind direction. These refer to the period of analysis 21–23 July 2021 that were the hottest days in the last summer. All the input meteorological features were homogeneous in the analyzed area; this type of input complies with the local approach of the study. The main time steps during the 72-h analysis were adapted to minimize spinning-up effects and to reach an equilibrium state. The software provided a dynamic adaptation of the main time step. The criterion for time step selection was the height of the sun. During the first 30 min of simulation, the model sets the main time step in order to improve numerical stability during the model spin-up phase. This methodology enables quickly convergent solutions to be obtained. The absolute differences between the results of the first and second simulation day were significant, while they were negligible between the second and third day.

**Figure 14:**
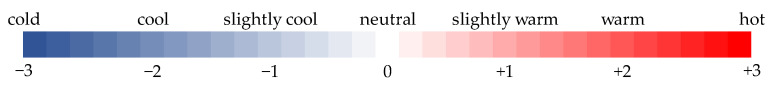
PMV Scale.

The ENVI-met application tool Leonardo [1] allowed a two-dimensional (2D) visualization of the output colored maps to compare the simulation scenarios [21] having regard to:

- air temperature (AT) in the examined square, and
- predicted mean vote (PMV) (i.e., a human comfort index that summarizes the impact of six parameters: air temperature, mean radiant temperature, wind speed, moisture, clothing and activity level) [42]. The PMV ranges between +/−3, according to the scale in Figure 14.
  The range +/−1 is the so-called comfort zone and represents the ideal condition. It must be emphasized that applying the PMV equation to outdoor conditions in summer heat stress situations can easily produce high PMV values above +3 (+5 and more).

## 3. Results

**Figure 15:**
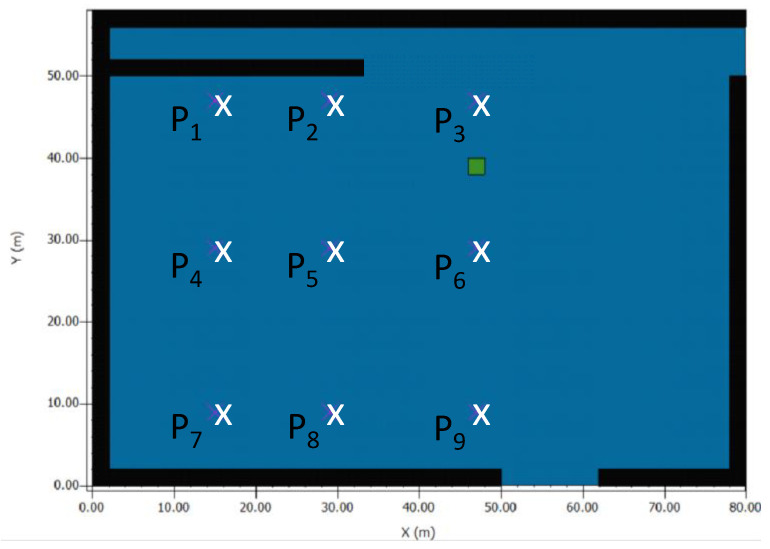
Layout of the ENVI-met model.

Figure 15 shows the position of 9 selected points (Pi, with i = 1, …, 9) in the modeled survey site; black pixels are buildings and walls surrounding the square and the green pixel (that is, in Figure 11, Figure 12 and Figure 13) is a tree.

Table 4 lists the air temperature and PMV values obtained for each scenario and surveyed point at 06:00 a.m. and 04:00 p.m. because at these hours AT reached the lowest and highest values at the hottest point (i.e., P3) during the third analyzed day.

Table 5 summarizes the minimum and maximum AT values calculated for the examined scenarios.

Table 6 summarizes the minimum and maximum PMV values calculated for the examined scenarios.

**Figure 16:**
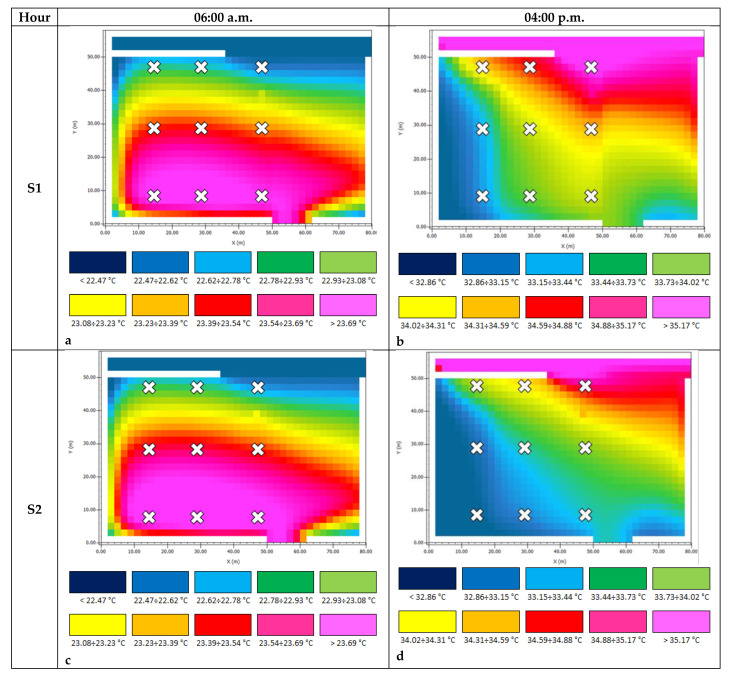

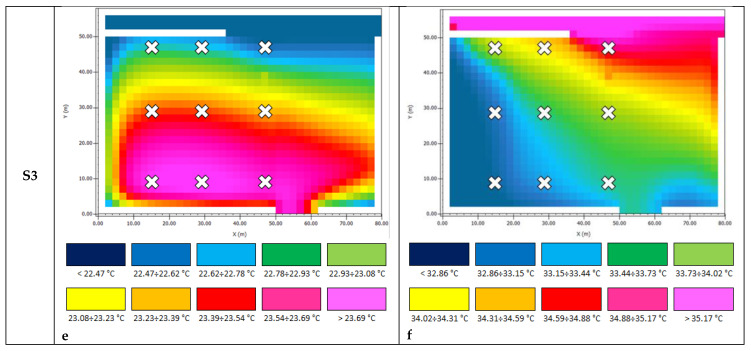
AT output maps. (**a**) S1 06:00 a.m.; (**b**) S1 04:00 p.m.; (**c**) S2 06:00 a.m.; (**d**) S2 04:00 p.m.; (**e**) S3 06:00 a.m.; (**f**) S3 04:00 p.m.

**Figure 17:**
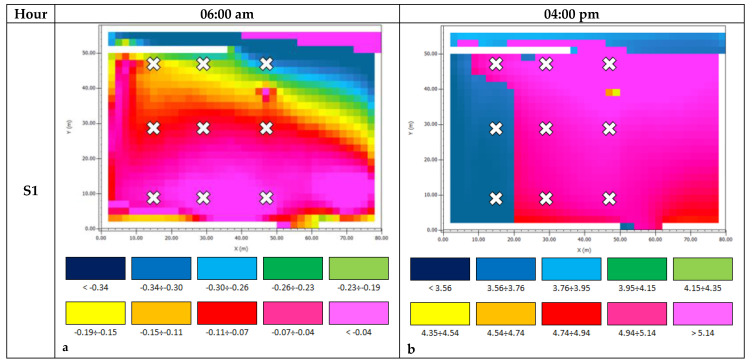

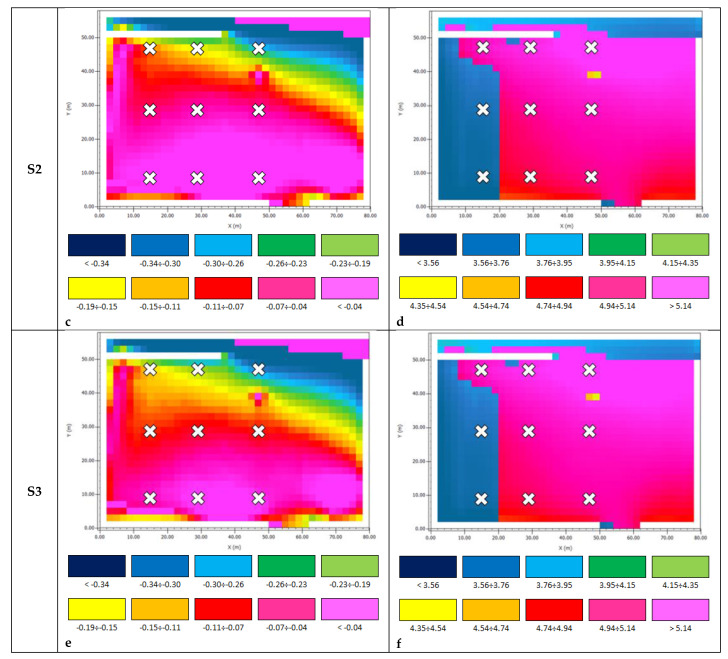
PMV Output maps. (**a**) S1 06:00 a.m.; (**b**) S1 04:00 p.m.; (**c**) S2 06:00 a.m.; (**d**) S2 04:00 p.m.; (**e**) S3 06:00 a.m.; (**f**) S3 04:00 p.m.

Figure 16a–f and Figure 17a–f show the AT and PMV chromatic output maps, respectively; the red crosses are the positions of Pi.

## 4. Discussion

At 6:00 a.m., the ATs (Figure 16a,c,e, Table 4 and Table 5) showed that cool pavements did not significantly alter the current scenario; temperature differences were not appreciable when asphalt pavement in S1 was substituted by sampietrini pavers with traditional and permeable patterns in S2 and S3, respectively. For the surveyed points, S3 was the coolest solution; its average AT value was 0.04 °C less than S1 and 0.11 °C less than S2, which was the hottest solution. On the other hand, cool pavements significantly affected AT day-time performances (Figure 16b,d,f, Table 4 and Table 5). The difference was appreciable, as shown in Figure 16b, where temperature discontinuities between adjacent pavements were noticeable. These results are consistent with the thermal properties of the pavement materials. In P6, P8, and P9, S2 had AT values at least 0.60 °C lower than S1. The permeable sampietrini pavement (i.e., S3) ensured an average AT reduction equal to 0.45 °C (the maximum benefit was in P6 where AT was 0.55 °C lower than the value obtained in S1).

Having regard to PMV (Figure 17a,c,e, Table 4 and Table 6) at 6:00 a.m., the obtained values were within the thermal comfort range discussed in the previous section. All scenarios had comparable PMV values, which ranged between −0.34 and 0.04. This result is consistent with the discussed trend of AT at the same time; the road pavement did not affect the microclimatic conditions. The day-time PMV values (Figure 17b,d,f, Table 4 and Table 6) highlight that the heat was unbearable in all the examined scenarios (the minimum value was 3.56 for P7 in S1). The differences between the PMV average values for all the solutions were not appreciable. The two scenarios implemented with the sampietrini pavements (i.e., S2 and S3) produced almost identical results, while the S1 values were a little higher than those of S2 and S3, except for P4 and P7.

The obtained results demonstrate that the compared pavement types did not influence the PMV values. Other strategies should be implemented to improve the climatic conditions (e.g., use of light materials to pave the road surface, or planting trees), where possible. In the analyzed area, unfortunately, urban constraints do not allow these alternatives.

## 5. Conclusions

In order to counter urban heat islands, many scientific studies have been carried out to identify the best mitigation methods. Increased use of green infrastructures (e.g., green roofs, green walls, green corridors, and green networks) and the replacement of existing pavements with cool ones (e.g., stone) are the most investigated solutions. Compared to traditional asphalt pavements, cool pavements lower the day-time surface temperature and reduce the amount of absorbed heat. They are composed of alternative materials, such as stone elements, or rely on new technologies, which increase the albedo value.

The proposed study sought to assess how replacement of existing asphalt with stone pavement might improve the microclimatic conditions of an urban area. This study focused on two cool pavements composed of sampietrini pavers that differed in their pattern layout; one was traditional, the other permeable. The software ENVI-met 3.4 LITE was used to carry out a three-dimensional microclimate analysis considering the surrounding environment (e.g., meteorological conditions, urban fabric, and vegetation). Three scenarios, involving different road pavement (i.e., existing asphalt, traditional sampietrini, and permeable sampietrini pavement), were modeled to characterize the St Peter in Chains’ square in Rome during summer conditions. Pavement performances throughout the day, in terms of air temperature and predicted mean vote, were compared to assess the effectiveness of black basalt pavers in preventing UHI. The comparison between the results revealed that the cobblestones pavement created a significant benefit in terms of reduction of day-time air temperature (the air temperature dropped by up to 0.6 °C). However, the benefit deriving from this approach was appreciable only during the day; in early morning the air temperature did not change compared to the current situation. As for the observed PMV values, the pavement type did not affect PMV.

Although the simulations focused only on the thermal and climate performance of the road pavement, the positive results obtained in terms of reduction of air temperature demonstrate that cool pavements could be part of a strategy to mitigate UHI. Therefore, surrounding buildings characteristics and green infrastructures should be considered to assess their effect on the urban microclimate environment.

## Figures and Tables

**Table 1:** Emissivity and albedo coefficients of building materials.

Material	Albedo	Emissivity
Granite	0.50	0.96
Limestone	0.70	0.93
Marble	0.80	0.95
Basalt	0.40	0.90
Concrete	0.50	0.85
Asphalt	0.10	0.93

**Table 3:** Input data in the ENVI-met simulation model.

Tmax (°C)	Tmin (°C)	Wind Speed (m/s)	Wind Direction (°)
35	21	3.0	West

**Table 4:** AT and PMV values at 06:00 a.m. and 04:00 p.m.

Scenario	Surveyed Point	AT (°C)	PMV	AT (°C)	PMV
		06:00 a.m.		04:00 p.m.	
S1	P1	22.78	−0.19	34.57	5.24
	P2	22.75	−0.25	34.92	5.33
	P3	22.51	−0.33	35.75	5.53
	P4	23.51	−0.08	33.42	3.61
	P5	23.52	−0.06	34.14	5.20
	P6	23.41	−0.07	34.55	5.29
	P7	23.88	−0.02	33.31	3.56
	P8	23.91	0.01	33.87	5.12
	P9	23.80	0.00	34.15	5.19
S2	P1	22.84	−0.17	34.12	5.19
	P2	22.81	−0.22	34.42	5.27
	P3	22.58	−0.30	35.22	5.46
	P4	23.58	−0.05	33.03	3.66
	P5	23.59	−0.03	33.58	5.12
	P6	23.50	−0.04	33.91	5.19
	P7	23.96	0.01	32.86	3.59
	P8	24.00	0.04	33.26	5.03
	P9	23.90	0.03	33.55	5.11
S3	P1	22.76	−0.20	34.18	5.21
	P2	22.72	−0.25	34.49	5.29
	P3	22.47	−0.34	35.30	5.49
	P4	23.48	−0.09	33.09	3.68
	P5	23.48	−0.07	33.65	5.14
	P6	23.37	−0.08	34.00	5.22
	P7	23.83	−0.03	32.93	3.62
	P8	23.87	0.01	33.35	5.06
	P9	23.76	−0.01	33.63	5.13

**Table 5:** Minimum and maximum AT values at 06:00 a.m. and 04:00 p.m.

Scenario	Minimum AT (°C)	Maximum AT (°C)	Minimum AT (°C)	Maximum AT (°C)
	06:00 a.m.		04:00 p.m.	
S1	22.51	23.91	33.31	35.75
S2	22.58	24.00	32.86	35.22
S3	22.47	23.87	32.93	35.30

**Table 6:** Minimum and maximum PMV values at 06:00 a.m. and 04:00 p.m.

Scenario	Minimum PMV	Maximum PMV	Minimum PMV	Maximum PMV
	06:00 a.m.		04:00 p.m.	
S1	−0.33	0.01	3.56	5.53
S2	−0.30	0.04	3.59	5.46
S3	−0.34	0.01	3.62	5.49

## Data Availability

The data presented in this study are available on request from the corresponding author. The data are not publicly available due to confidentiality reasons.
